# Effect of a One-Time Communication Training Session on Dental Students’ Self-Efficacy Expectancy

**DOI:** 10.3390/ijerph20043323

**Published:** 2023-02-14

**Authors:** Thekla J. Grötz, Lisa Zöll, Anke Hollinderbäumer, Thomas Nowak, Tamara Zajontz, Lina Behling, Sabine Fischbeck, Uwe Schmidt, James Deschner

**Affiliations:** 1 Department of Periodontology and Operative Dentistry, University Medical Center, Johannes Gutenberg University, 55131 Mainz, Germany; 2 University Medical Center, University of Mainz, Rudolf Frey Lernklinik, 55131 Mainz, Germany; 3 Center for Quality Assurance and Development (ZQ), Johannes Gutenberg University Mainz, 55124 Mainz, Germany; 4 Department of Psychosomatic Medicine and Psychotherapy, Medical Psychology and Medical Sociology, University Medical Center, Johannes Gutenberg-University, 55099 Mainz, Germany

**Keywords:** communication skills, dental curriculum, dental education, self-efficacy expectancy

## Abstract

An implementation of training units that provide evidence for improving students’ communication skills in the dental curriculum is now more than ever of utmost importance. This study aimed to investigate how students assess their skills after communication training and whether this training also increased students’ self-efficacy expectancy. A total of 32 male and 71 female students with a mean age of 25.6 ± 3.9 years participated in the study. Self-assessment of communication skills and self-efficacy expectancies were collected at two time points using Likert scales. Our study shows that the communication training, consisting of a practical exercise with actors and an online theory module, significantly improved the students’ self-assessment of their communication skills and also improved some aspects of self-efficacy expectancy. These results indicate that, in addition to the practical and technical-theoretical training of students, communication training is essential in the dental curriculum. In summary, this study showed that a one-time practical exercise with actors together with an online theory module could improve both the self-assessment of communication competence and some aspects of self-efficacy expectancy, which demonstrates the importance of training communication skills alongside practical and technical-theoretical training.

## 1. Introduction

Dental visits are perceived as stressful by a majority of patients. In a representative study, around 60% of the study population experienced at least some fear during dental visits, while 10% suffered from a diagnosed dental phobia and avoided dentals visits [1]. Delayed or avoided dental visits worsen oral hygiene and predispose patients for a poorer dental status. Despite this fear mostly resulting from previous painful dental experiences, patients primarily desired accurate information about their dental treatment and a compassionate dentist, suggesting that according communication might benefit these patients [1]. This proves that appropriate communication is beneficial for these patients [2,3]. Good communication, both verbal and non-verbal, creates a basis of trust between patient and dentist, and significantly influences the subsequent treatment and thus the success of the treatment.

In dentistry, communication takes place both verbally and non-verbally in the dentist–patient conversation, before and after treatment. As a special situation, it should be emphasized that during dental treatment, the patient has only a limited possibility to communicate, while the practitioner can communicate freely. It should also be mentioned that the success of the treatment depends only little on the manual skills of the dentist, but is significantly influenced by the communication [4,5]. A structured communication is very important for both the dentist and the patient [6]. The advantages of successful communication for the patient are increased satisfaction, improved therapeutic results and a reduction in the recourse rate [7,8,9,10]. In addition, good communication increases the satisfaction of the dentist [11]. This has been shown to reduce burnout in the dental profession [12,13].

Although some universities have some training sessions for acquiring communication skills, these are mostly for medical students only. Even where dental students also receive communication training, there is usually no assessment of learning [14,15]. Moreover, content varies from university to university. However, teaching communication skills is highly appreciated by students, dentists and also patients [16]. 

Since 2020, patient dental education has changed significantly due to the SARS-CoV-2 pandemic. The transition to digital teaching and the cancellation of patient treatments have further limited the opportunities and possibilities for students to acquire communicative skills. An implementation of training units for the acquisition of communicative skills in the dental curriculum is therefore currently more than ever of utmost importance. Nevertheless, such training must also provide evidence that students’ communicative skills improve as a result of the intervention.

Therefore, the aim of this study was to investigate how students rated their skills after the communication training and whether this training also increased students’ self-efficacy expectancy.

## 2. Materials and Methods

### 2.1. Sample Composition

For the study, students in the first and last clinical semesters, i.e., the 7th and 10th semesters, of the dental curriculum at Johannes Gutenberg University Mainz were surveyed from October 2020 to February 2021. In both semesters, students were divided into an intervention group (IG) and a control group (CG). A total of 103 students participated in the study. The IG included 51 students, while the CG comprised 52 students.

### 2.2. Intervention and Control Groups

Here, an online theory module, a unit of face-to-face teaching and a video-based training session were carried out. An evaluation of the self-assessed learning success was carried out by means of questionnaires, which were completed by the students before and after the module. 

At the beginning of the semester, the students received a questionnaire for self-assessment of their communicative skills, which was filled out pseudonymously (time T0). Subsequently, a lecture was made available in the existing online learning platform for self-study (theory module). In the third week of the semester, the practical unit of the communicative skills classroom teaching took place. In this practical module, the students conducted conversations with acting patients who portrayed different characters. In the fifth and sixth week of the semester, the questionnaire for self-assessment of communicative skills was distributed again as a pseudonymized questionnaire (time T1). This made it possible to compare the self-assessment from T0 to T1 in both groups. The control group received no interventions or information between T0 and T1. Only after T1 were they provided with the theory module and the practical teaching unit. 

### 2.3. Online Theory Module

The online theory module provided a professional background on communication techniques in the form of a PowerPoint presentation given by two dentists (T.J.G, L.Z.). The online theory module lasted 45 min twice. For this online module, the following learning objectives of the National Competence-Based Learning Objectives Catalog for Dentistry (NKLZ), which describes the intended profile of dental school graduates in Germany, were used as a basis:

(a) Students can establish and maintain a positive, sustainable and trusting dentist–patient relationship through their communicative actions [3].
(b) Students can structure patient conversations from beginning to end. They can weigh the parts differently depending on the type of conversation [3].
(c) Students know the importance of non-verbal communication and use positive, non-verbal signals [3].
(d) Students are able to apply appropriate interview and questioning techniques as appropriate to the situation [3].
(e) Students can deal adequately with emotionally challenging situations in the context of the dentist–patient relationship [3].

The lecture was developed with the Centre for Quality Assurance and Development and the Department of Medical Psychology and Medical Sociology at the University Medical Center Mainz.

### 2.4. Practical Teaching Unit

In the practical training for the intervention group, the students were confronted with a standardized educational situation. The practical module was developed together with the Rudolph Frey Learning Clinic. Actors were available who had already worked with similar educational activities as part of the communication module in the human medicine program. All actors were assigned the same initial situation, and each was given a role in the form of a character trait. The initial situation for each role was a patient who had already been seen in the outpatient clinic and had been diagnosed with periodontitis. The interview to be conducted represented the consultation and explanation of a periodontitis treatment. All the characters portrayed had diabetes type 2 as an underlying disease. The actors portrayed the following characters:

- An anxious patient;
- A patient annoyed after a long waiting time;
- A patient over-informed by the internet;
- A patient with possible HIV infection;
- A frequent talker;
- A quiet patient who is difficult to approach.

These characters gave the opportunity to simulate emerging conflict situations. Each student had the opportunity to work on at least one case study. Afterwards, the students received feedback from the acting patients, the assistant doctor and their fellow students. Each character was video-recorded as a case study with the consent of the respective student.

The control group watched the practical training of the intervention group in the video-based lesson. This was followed by a discussion of the situation in plenary.

At the end of the semester, the students received the self-assessment questionnaire again, which could be assigned to the previously completed questionnaire through pseudonymization.

The questionnaire for recording the self-assessed communication skills was developed together with the Centre for Quality Assurance and Development and then transferred to a survey software (EvaSys, Electric Paper Evaluationssysteme GmbH, Lüneburg, Germany). This enabled the questionnaires to be evaluated electronically. In order to pseudonymize the questionnaires and then reassemble them anonymously, a personal code was created for each student. This code consisted of the date of birth and the names of the parents and the student and was entered on the respective questionnaire. The code was known only to the student. The questionnaire collected socio-demographic data, the students’ self-assessment of their communication skills and their self-efficacy expectancy.

### 2.5. Sociodemographic Parameters

The following socio-demographic parameters were collected: Age, gender and mother tongue. 

#### 2.5.1. Assessment of Communication Competence

Ten statements were formulated to assess the participants’ own communication competence, based on the learning objectives:

1.1: I am able to build up a positive and trusting relationship with the patient.

1.2: I involve the patient in the decision-making process.

1.3: I structure the patient conversations. 

1.4: I use positive signals of non-verbal communication. 

1.5: I can summarize information for the patient. 

1.6: I respond to the patient’s emotions. 

1.7: I can deal with different behaviors of the patient. 

1.8: I can use questioning techniques adequately. 

1.9: I have achieved my treatment goal on today’s treatment day. 

(The question was deleted because no treatment was carried out due to SARS-CoV-2 pandemic). 

1.10: I give the patient the opportunity to describe his or her own concerns.

The questions could be answered using a five-point Likert scale from ‘strongly disagree’ (1) to ‘strongly agree’ (5).

#### 2.5.2. Self-Efficacy Expectancy

The Schwarzer and Jerusalem scale was used to assess the change in the students’ general self-efficacy expectancy [17]. The 10 questions given in the validated scale could be answered with a four-point Likert scale from “not true” (1) to “true exactly” (4).

2.1: When resistance arises, I find ways and means to assert myself.

2.2: I always succeed in solving difficult problems when I try.

2.3: I have no difficulty in realizing my intentions and goals.

2.4: In unexpected situations I always know how to act.

2.5: Even when faced with surprising events, I believe I can cope well.

2.6: I face difficulties calmly because I can always trust my abilities.

2.7: Whatever happens, I will manage.

2.8: I can find a solution to any problem.

2.9: When a new thing comes my way, I know how to deal with it.

2.10: When a problem arises, I can cope with it on my own.

### 2.6. Statiscal Analysis

Statistical analysis and presentation of the data was performed using SPSS Statistics 23 (IBM, Armonk, New York, NY, USA) and Excel version 2102 (Microsoft, Redmond, Washington, DC, USA) and was supervised by the Institute of Medical Biometry, Epidemiology and Informatics (IMBEI) of the University Medical Center Mainz. A descriptive analysis of the socio-demographic data was carried out. The other data collected were analyzed using a *t*-test. Mean comparisons of the two groups were made using a paired-samples *t*-test because a normal distribution could be assumed. The significance level was set at *p* < 0.05 in each case.

## 3. Results

Thirty-two male and 71 female students participated in the study. The average age of the students was 25.6 ± 3.9 years. In addition, 83 of the students reported German and 20 reported another language as their native language. 

### 3.1. Assessment of Communication Competence

**Figure 1:**
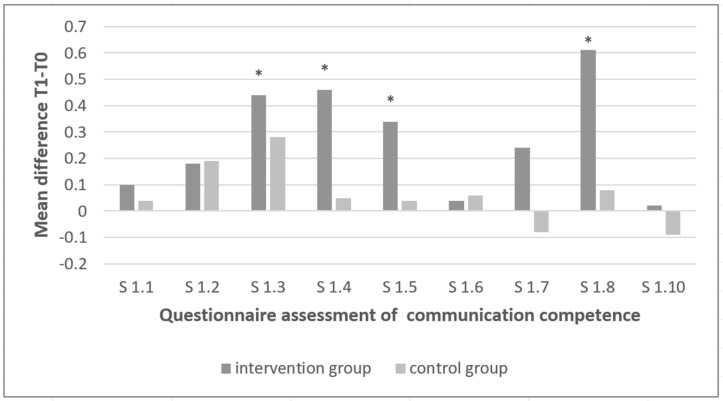
Questionnaire assessment of communication competence. (* significant at *p* < 0.05).

Our study showed a significant improvement for the self-assessment of communication skills in the intervention group from time T0 to T1 for the sum of all statements (*p* = 0.001) and for the single statements 1.3 (I structure the patient conversations, *p* = 0.011), 1.5 (ability to summarize information for the patient, *p* = 0.002), 1.4 (I use positive signals of non-verbal communication, *p* = 0.003) and 1.8 (I can use questioning techniques adequately, *p* = 0.000) (Figure 1). 

In contrast, within the control group, there were no significant differences between the two time points for any of the statements. In the control group, there was a trend toward worsening with respect to the statements 1.7 (I can deal with different behaviors of the patient, *p* = 0.935) and 1.10 (I give the patient the opportunity to describe his or her own concerns, *p* = 0.735) at T1 compared with T0 (Figure 1). 

### 3.2. Self-Efficacy Expectancy

**Figure 2:**
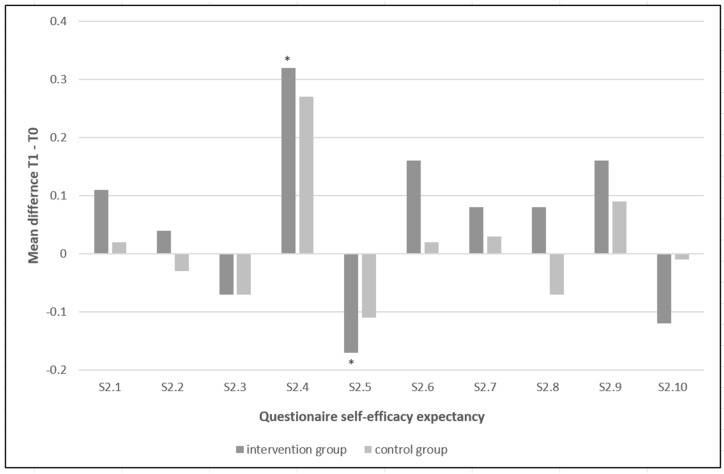
Questionnaire self-efficacy expectancy. (* significant at *p* < 0.05).

Our analysis of the general self-efficacy expectancy revealed no significant differences between T0 and T1 in the intervention group when all statements were considered together. However, significant differences between T0 and T1 in the intervention group were found for the self-efficacy for the statements 2.4 (I always know how to act in unexpected situations, *p* = 0.018) and 2.5 (I cope well even when faced with surprising events, *p* = 0.015) (Figure 2). The intervention group showed a significant improvement when assessing their ability to deal with unexpected situations in statement 2.4 but also a significantly worse result in the similar statement 2.5 (Figure 2).

In contrast, within the control group, there were no significant differences between the two time points, either for all statements combined or for any of the statements alone (Figure 2).

## 4. Discussion

Our study shows that a communication training consisting of a practical exercise with actors and an online theory module significantly improves self-assessment of communication competence in students. Furthermore, the communication training was also able to improve some aspects of self-efficacy expectancy. These results suggest that in addition to the practical and technical-theoretical training of students, communication training is essential in the dental curriculum. 

It could be shown that the self-assessment of communicative skills experienced a significant improvement as a result of the one-time communication training. The intervention group showed a significant increase in self-assessment after the training. In contrast, there was no significant change in the control group. In the intervention group, all self-assessment statements improved, with statistically significant improvements for all statements together and the statements 1.3 (I structure the patient conversations), 1.4 (I use positive signals of non-verbal communication), 1.5 (I can summarize information for the patient), and 1.8 (I can use questioning techniques adequately). Although not exclusive, the improvement in statements 1.3 and 1.5 could be attributed to the Calgary–Cambridge concept [18,19]. The Calgary–Cambridge concept describes the dental interview structure and is considered comprehensible and easy to integrate into everyday clinical practice [18,19]. The statements that showed improvement but were not statistically significant could be related to the more complex skills that were asked in these statements. The fact that these more complex skills were not optimally developed could be that because of the SARS-CoV-2 pandemic, the treatment of patients was partially restricted and, therefore, the learned knowledge could not be fully applied. Interestingly, there was a trend toward deterioration in the control group with respect to statements 1.7 (I can deal with different behaviors of the patient) and 1.10 (I give the patient the opportunity to describe his or her own concerns) between the beginning and the end of the study. The fact that the outcomes worsened without communication training, even though only tendentially, emphasizes the potential of a one-time communication training not only to further develop skills of the students, but even to compensate for inhibiting factors.

In our study, students’ communication training had no significant effect on overall self-efficacy expectancy when all statements were considered together. This reflects a stable personality trait of the students, especially in everyday situations [20]. The self-efficacy expectancy scale was formulated very generally, and it should be emphasized that there was no strong reference to communication. The students had already indicated a very high self-efficacy expectancy at the beginning of their study program. The students in our study were already at a higher semester of their program and therefore already had a high self-efficacy expectancy. Interestingly, when the statements were considered separately, significant differences were evident for statements 2.4 (I always know how to act in unexpected situations) and 2.5 (I can cope well even when faced with surprising events). Statements 2.4 and 2.5 deal with the handling of unexpected situations. Among the students who participated in the communication training, there was a significant improvement for them regarding statement 2.4, but a significant worsening for statement 2.5. These contradictory statements by the students after the communication training are surprising, since these statements are basically similar in content. This suggests that statement 2.5 may have been misunderstood by the students. In contrast, no significant differences were found in the control group between the two time points, either for all statements together or for any of the statements alone. Regarding the self-efficacy expectancy, it would be desirable to have a scale that not only refers to general aspects, but also to the patient treatment and the clinical section of the dental curriculum.

The ratio of female to male students was 2:1 in our study. This corresponds to the normal gender distribution in dental schools in Germany. It would be interesting to know if the results of this study would be different for an all-female and all-male student cohort. Future studies with even larger numbers of participants could address this relevant question. The mean age of the students in our study was approximately 26 ± 4 years, indicating that a broader age range was covered in our study. Although some students did not report German as their native language, all students were highly proficient in German, as their dental program was also entirely in German.

A limitation of our study was that only a self-assessment by the student took place. It would also be interesting to have a 360° evaluation, in which the different aspects of communication competence after such communication training are not only assessed by the student himself, but also by the dental instructor, the assisting student and the patient. Such an evaluation from different perspectives would allow a more objective assessment of the potential of a communication training.

## 5. Conclusions

In summary, this study showed that a one-time practical exercise with actors together with an online theory module was able to improve both self-assessment of communication competence and some aspects of self-efficacy expectancy in students, suggesting that in addition to practical and professional theoretical teaching, training in communication skills is of paramount importance.

## Data Availability

Data chairing is not applicable to this article.
